# Targeting Eukaryotic Translation in Mesothelioma Cells with an eIF4E-Specific Antisense Oligonucleotide

**DOI:** 10.1371/journal.pone.0081669

**Published:** 2013-11-18

**Authors:** Blake A. Jacobson, Saritha C. Thumma, Joseph Jay-Dixon, Manish R. Patel, K. Dubear Kroening, Marian G. Kratzke, Ryan G. Etchison, Bruce W. Konicek, Jeremy R. Graff, Robert A. Kratzke

**Affiliations:** 1 Department of Medicine, University of Minnesota, Minneapolis, Minnesota, United States of America; 2 Department of Biological Sciences, University of Wisconsin-Fox Valley, Menasha, Wisconsin, United States of America; 3 Research Service, Minneapolis Veterans Affairs Medical Center, Minneapolis, Minnesota, United States of America; 4 Lilly Research Laboratories, Eli Lilly and Company, Indianapolis, Indiana, United States of America; Korea University, Korea, Republic Of

## Abstract

**Background:**

Aberrant cap-dependent translation is implicated in tumorigenesis in multiple tumor types including mesothelioma. In this study, disabling the eIF4F complex by targeting eIF4E with eIF4E-specific antisense oligonucleotide (4EASO) is assessed as a therapy for mesothelioma.

**Methods:**

Mesothelioma cells were transfected with 4EASO, designed to target *eIF4E* mRNA, or mismatch-ASO control. Cell survival was measured in mesothelioma treated with 4EASO alone or combined with either gemcitabine or pemetrexed. Levels of eIF4E, ODC, Bcl-2 and β-actin were assessed following treatment. Binding to a synthetic cap-analogue was used to study the strength of eIF4F complex activation following treatment.

**Results:**

eIF4E level and the formation of eIF4F cap-complex decreased in response to 4EASO, but not mismatch control ASO, resulting in cleavage of PARP indicating apoptosis. 4EASO treatment resulted in dose dependent decrease in eIF4E levels, which corresponded to cytotoxicity of mesothelioma cells. 4EASO resulted in decreased levels of eIF4E in non-malignant LP9 cells, but this did not correspond to increased cytotoxicity. Proteins thought to be regulated by cap-dependent translation, Bcl-2 and ODC, were decreased upon treatment with 4EASO. Combination therapy of 4EASO with pemetrexed or gemcitabine further reduced cell number.

**Conclusion:**

4EASO is a novel drug that causes apoptosis and selectively reduces eIF4E levels, eIF4F complex formation, and proliferation of mesothelioma cells. eIF4E knockdown results in decreased expression of anti-apoptotic and pro-growth proteins and enhances chemosensitivity.

##  Introduction

 Malignant mesothelioma, which is responsible for the deaths of 3000 Americans annually, is not curable with existing therapies [1]. The current standard of care for unresectable mesothelioma is the combination of cisplatin and pemetrexed that leads to a median time to progression of 7 months and overall survival of 12 months [2]. More effective therapeutic strategies are necessary for this fatal disease.

In eukaryotes, newly produced transcripts are modified by the addition of a 7-methylguanosine cap at their 5’ end. In the cytoplasm, eIF4E is the cap-binding protein component of the eIF4F complex that is also comprised of the RNA helicase eIF4A and the scaffolding protein eIF4G. Once assembled the eIF4F complex scans, 5’-3’, through the 5’ untranslated region (UTR) while unwinding mRNA secondary structure toward the translation initiation codon that enables translation. The availability of eIF4E is considered to be rate limiting for the assembly of the eIF4F complex [3,4]. Under normal physiologic conditions the eIF4E binding proteins (4E-BP) negatively regulate translation initiation by sequestering eIF4E from eIF4G. During permissive growth conditions 4E-BP1 is preferentially phosphorylated by the Ras/phosphoinositide 3-kinase (PI3K)/AKT/mammalian target of rapamycin (mTOR) kinase cascade. Phosphorylation of 4E-BP1 lessens the affinity of 4E-BP1 for eIF4E allowing eIF4E to bind to eIF4G permitting eIF4F assembly and driving cap-dependent translation [5–7].

The assembly of the eIF4F complex is dependent on the availability of active eIF4E. When 4EBP1 is phosphorylated, eIF4E is released from 4EBP1 allowing binding to eIF4G, enabling eIF4F assembly resulting in cap-dependent translation. “Strong” mRNAs are efficiently translated but “weak” mRNAs, which have longer 5’ UTR sequences and complex secondary structures require elevated eIF4F activity. In cancer, eIF4F potency is enhanced either by an increase in eIF4E expression or by cell signaling through PI3K/AKT/mTOR pathway or by both. This consequently enables a disproportionate increase in the translation of those “weak” mRNAs, many of which encode malignancy-related genes that are involved in cell growth, cell survival or angiogenesis. Thus, subsets of mRNAs from different oncogenic pathways that contribute to tumorigenesis are selectively activated [8,9]. In fibroblasts and primary epithelial cells overexpression of eIF4E was sufficient to induce transformation [10,11]. Additionally, surveys of many different human cancer types indicate that eIF4E levels are elevated in malignancies of the prostate, lung, breast, stomach, colon, skin as well as cancers of the hematopoietic system [12–18]. Considering this evidence, eIF4E can be regarded as an oncogene that represents an attractive therapeutic target which functions at the intersection of cellular pathways controlling malignancy.

With this in mind therapies that target the deranged cap-dependent translation engendered by eIF4E hyperactivation have been explored. In murine xenograft models of breast [11] and lung cancer [13] eIF4E inhibition by ectopic overexpression of an activated 4EBP1 led to abrogated tumorigenicity. Similarly, data suggests that mesothelioma is reliant upon cap-dependent translation pathways. In mesothelioma cell lines, evidence indicates constitutive activity of cap-dependent translation [19]. Furthermore, inhibition of cap-mediated translation by overexpression of a phosphorylation-defective 4EBP1 virtually eliminates tumorigenicity of mesothelioma cell lines [20].

Other studies that employ short-interfering RNA against eIF4E [21,22], peptides directed to the eIF4E - eIF4G interaction site [23,24], small molecule inhibition of eIF4E - eIF4G interaction [25,26] and pharmacological inhibition of eIF4E - mRNA cap interaction [27] have been investigated. These approaches of directly targeting eIF4F integrity and function by eIF4E antagonism provide evidence of therapeutic efficacy in each cancer studied. 

Selective targeting of *eIF4E* mRNA by specific antisense oligonucleotide (ASO) therapy has also been studied in cancer [28]. eIF4E ASO (4EASO) is a second generation ASO that specifically targets *eIF4E* mRNA for destruction and is engineered for enhanced nuclease resistance and potency. Preclinical use of this 4EASO elicited single agent activity in human cancer xenografts (breast, prostate), decreasing eIF4E expression and diminishing tumor growth without toxicity [28]. When prostate cancer [12] and non-small cell lung cancer cell lines (manuscript in preparation) were exposed to 4EASO a dose-dependent inhibition of cancer cell survival was observed. The success of these investigations led to a phase 1 clinical trial of 4EASO (LY2275796) in human cancer that clearly demonstrated reduction of eIF4E mRNA and protein within patient tumor tissues [29].

In this study, 4EASO that specifically targets *eIF4E* mRNA for destruction is assessed as a therapeutic agent against mesothelioma. 4EASO potently diminished eIF4E protein levels, repressed cap-dependent complex formation, selectively reduced eIF4E regulated proteins (ODC and Bcl-2), reduced mesothelioma cell viability, induced apoptosis, and sensitized mesothelioma cells to gemcitabine and pemetrexed. 

## Materials and Methods

### Cell lines and Cell culture

The medium for mesothelioma cell lines, H2373, H2461 and H2596 (American Tissue Culture Collection) was RPMI 1640 (Gibco, Invitrogen) containing 10% calf serum (Biofluids). LP9 cells, non-transformed human mesothelial cells (National Institute on Aging Cell Repository), were cultured in a medium containing a 1:1 ratio of M199 and MCDB10 basal medium (Sigma) supplemented with 15% calf serum [not heat inactivated], 2mM glutamine, 10 ng/mL EGF and 0.4 μg/mL hydrocortisone. All cells were maintained at 37°C in 5% CO_2_.

### Antisense oligonucleotide transfection

The second-generation antisense oligonucleotides (ASOs) were provided by Eli Lilly and Company (Indianapolis, Indiana) and were 20 nucleotides in length [28]. 4EASO (LY2275796) has the sequence, 5’-TGTCATATTCCTGGATCCTT-3’, where the underlined nucleotides are 2’-methoxyethyl-modified (MOE) bases. It was designed to target the 3’ untranslated region of both the human and murine *eIF4E* mRNA. The mismatch ASO (mmASO) control has the sequence 5’-TCTTATGTTTCCGAACCGTT-3’ containing the same base composition as 4EASO. Oligofectamine (Invitrogen) was employed following the manufacturer’s instructions for ASO transfection. Briefly, Opti-MEM I (Gibco) and Oligofectamine (1:16 ratio) was mixed and incubated for seven minutes at room temperature. A mixture of 4EASO or mmASO combined with Opti-MEM I, to produce the desired final concentrations, was added to the Opti-MEM I and Oligofectamine solution and incubated together for 45 minutes. The cells were rinsed and fresh RPMI 1640 was deposited onto the cells followed by addition of the transfection solution (1:4 ratio) with subsequent incubation for 4 hours. Next, RPMI 1640 containing serum was added such that the final concentration of serum was 10% and the cells were either harvested or counted (by trypan blue exclusion employing a hemacytometer) 48 or 72 hours, respectively, later. For the preparation of cell lysates, 10 cm plates were used and 1 x 10^6^ cells were seeded one day prior to ASO transfection. 1.25 x 10^5^ cells were seeded into each well of six well plates for cell proliferation studies. The proliferation experiments were performed in triplicate.

### 4EASO treatment combined with either gemcitabine or pemetrexed

H2373 and H2596 cells were transfected as outlined above using 300 nM or 100 nM ASO [mmASO and 4EASO], respectively, and incubated overnight. The following day cells were treated with the indicated concentration of gemcitabine (Eli Lilly) and 48 hours later the cells were counted employing a hemacytometer coupled with trypan blue exclusion. For the ASO treatment combined with pemetrexed (Eli Lilly), the pemetrexed was added [10 nM for H2373 and 25 nM for H2596] the same day but following the treatment with ASO and cell number was determined 72 hours later. Cell survival is expressed as cell number normalized to untreated cells. Each experiment was performed in triplicate. Results are expressed as the mean +/- standard deviation. Group comparisons were done using two-sided Student’s *t*-test. Differences were considered significant at *P* <0.05. For the experiments utilized for combination index (CI) determination 5 x 10^3^ cells were seeded in the presence of 1% serum in each well of 96 well plates. Following overnight incubation, cells were transfected with ASO as above and 4 hours later serum replete medium (10%) containing pemetrexed or gemcitabine was added to achieve the desired final concentration of each to the cells. Cells were treated with varying concentrations of each drug alone and with four different combinations. Each of the treatment conditions were done on the same day using the same parent cells. The cells were then incubated for 72 hours at 37^O^C. Cell viability was determined by Cell Counting Kit-8 (Dojindo Molecular Technologies). Tetrazolium substrate was added to each well, plates incubated for 2 hours at 37^O^C, and absorbance measured at 450 nm. Cell viability values were normalized to untreated cells. Experiments were performed in triplicate. The degree of cooperation between 4EASO and pemetrexed or 4EASO and gemcitabine was determined using the Chou-Talalay method utilizing CompuSyn software. The resulting combination index (CI) quantitatively depicts synergism (CI<1), additive effect (CI=1) and antagonism (CI>1) [30].

### Cell lysate preparation

The cells were removed from the plates by scraping after washing once with PBS, after which cells were collected by centrifugation (14K rpm, 14 seconds), washed again with ice cold PBS followed by another round of centrifugation and resuspended in a volume of freeze-thaw lysis buffer (50 mM Tris-HCl pH 7.5, 150 mM NaCl, 50 mM NaF,1 mM EDTA, 10 mM tetrasodium pyrophosphate) supplemented with a protease inhibitor cocktail (Roche) and a phosphatase inhibitor cocktail (Sigma). The protein concentration of the lysates were determined following three freeze-thaw cycles (15 min at -80°C, 2 minutes at 37°C) by Bradford assay (Biorad) and stored at -80° C. In some instances the cells were resuspended in TNESV lysis buffer (50 mM Tris-HCl, pH 7.4; 1 % NP-40; 2 mM EDTA, pH 8.0; 0.1 M NaCl) containing the same protease and phosphatase inhibitors.

### *In vitro* cap-affinity assay

The strength of cap-mediated complex formation was measured as before [13]. Lysate (300 μg) from ASO treated and untreated cells were diluted in 300 μL freeze-thaw lysis buffer and mixed with 50 μL of a 50% slurry of 7-methyl GTP-Sepharose^TM^4B (Amersham Biosciences) and incubated while mixing for two hours at 4°C. Freeze-thaw lysis buffer containing 100 μM 7-methylguanosine 5’-triphosphate (Sigma-Aldrich) was employed to elute the captured eIF4E from the 7-methyl GTP-Sepharose^TM^4B beads and the eluted sample prepared for immunoblot analysis.

### Immunoblot analysis

Protein samples were separated by either 10 or 12% SDS-PAGE (polyacrylamide gel electrophoresis) except that the cap-affinity assay samples were separated by 8-15% gradient gels. Following protein transfer to PVDF (GE Healthcare) the membranes were blocked in 5% non-fat dry milk for 1 hour at room temperature in Tris-buffered saline-Tween (TBST: 0.15 M NaCl; 0.01 M Tris-HCl, pH 7.6; 0.05% Tween 20). The membranes were then incubated for 1 hour at ambient temperature or overnight at 4° C with the chosen primary antibody. The primary antibodies employed were rabbit α-eIF4E antibody (Cell Signaling), rabbit α-Bcl-2 antibody (Cell Signaling), mouse α-ODC antibody (Thermo Scientific), rabbit α-PARP (Cell Signaling) all at a 1:1000 dilution, and mouse α-β-actin (Sigma) at a 1:10,000 dilution. Preceding and following incubation with the appropriate horseradish peroxidase labeled secondary antibodies (SouthernBiotech), the blots were washed three times for 5 minutes in TBST and detection was then performed utilizing ECL Plus Western Blotting System (Amersham Biosciences) to visualize the bands of interest. The density of protein bands was determined using ImageJ, a public domain Java image processing program.

## Results

### 4EASO represses mesothelioma proliferation

**Figure 1:**
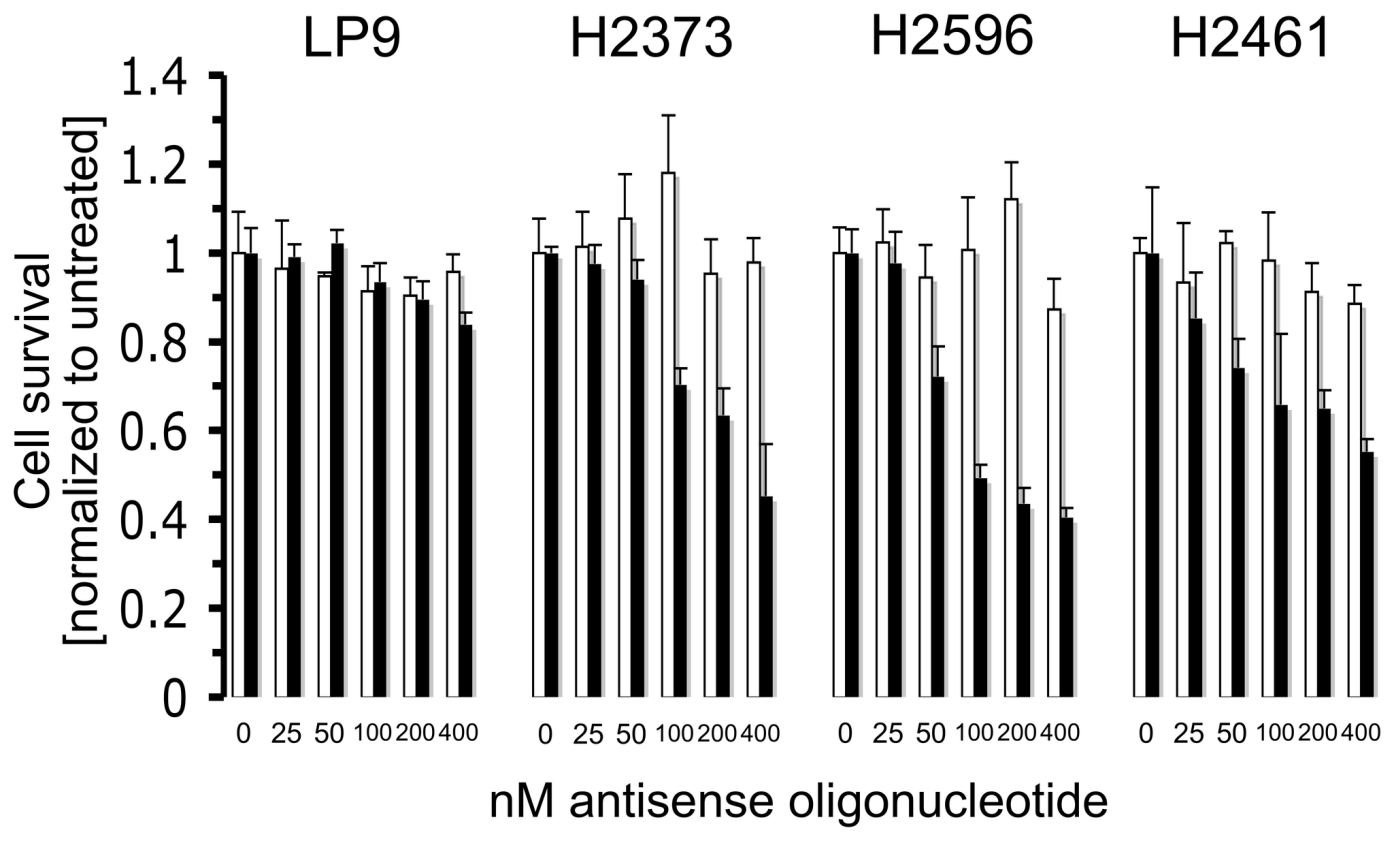
4EASO transfection suppresses mesothelioma proliferation. Normal mesothelial cells (LP9) and mesothelioma cell lines H2373, H2461 and H2596 were treated with the indicated concentration of mmASO (open columns) and 4EASO (filled columns), and viable cells were counted. Normal LP9 cells were most resistant to 4EASO treatment, while growth of mesothelioma cell lines was reduced extensively. *Columns*, the mean of three independent determinations of cell number normalized to untreated cells, *bars*, s.d.

In previous work it was shown that cap-mediated translation is enhanced in mesothelioma compared to normal mesothelial (LP9) cells and may be a consequence of Ras signaling pathway, phosphorylation of eIF4E and lower 4EBP1 levels [19]. In addition, the importance of deranged cap-dependent translation in mediating the effects of the IGF-I axis in mesothelioma was also established. Expression of a dominant active 4EBP1 resulted in decreased mesothelioma tumorigencity *in vitro* and in a murine xenograft mouse model [20]. These results demonstrate that the malignant phenotype conferred upon mesothelioma by aberrant cap-dependent translation may be abrogated by reduction of eIF4E activity. On the basis of these findings mesothelial and mesothelioma cells were transfected with a range of 4EASO concentrations and cell survival was assessed. Cell viability following 72 hours of treatment with 4EASO (Figure 1) demonstrated a dose-dependent effect on all cell lines and normal mesothelial cells. At the highest dose of 4EASO (400 nM) mesothelioma survival was between ~40-50%. The normal LP9 mesothelial cells were minimally impacted by 4EASO with a survival of ~85% at the highest dose. An alternative explanation for the differences in sensitivity between LP9 and the mesothelioma cells may be due to inherent transfection efficiencies or oligofectamine optimization differences between cell types [31,32]. The mmASO had a minor antiproliferative effect (~10%) on the cells at the highest concentration indicating that mesothelial and mesothelioma cells are not very sensitive to transfection with non-targeted, second generation ASOs. Of note, both epithelioid (H2461) and sarcomatoid (H2373 and H2596) derived mesothelioma cells were used in these assays [33]. Thus, 4EASO inhibited growth in different mesothelioma subtypes with nearly equal potency (Figure 1). 

### 4EASO treatment of mesothelioma diminishes eIF4E protein levels

**Figure 2:**
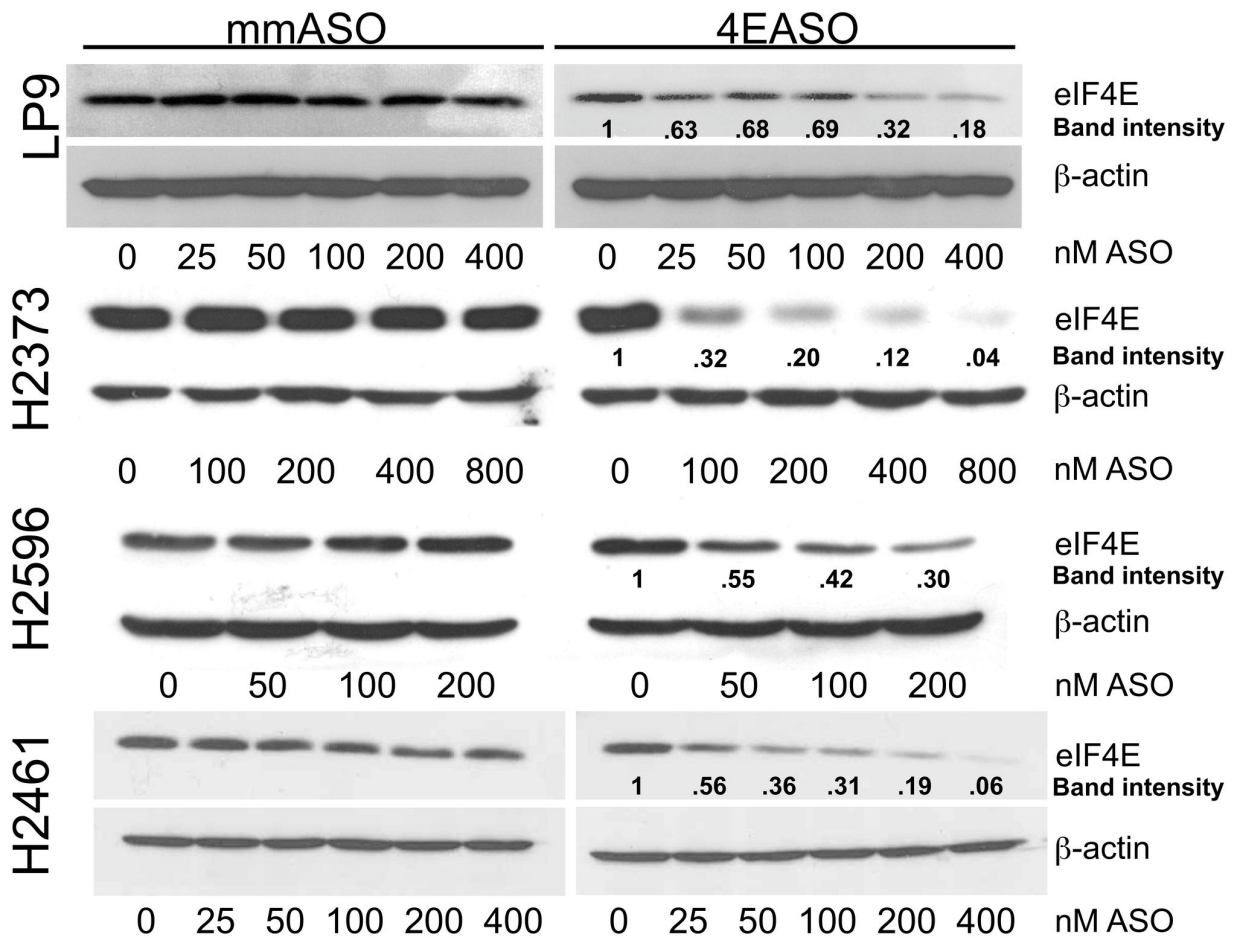
eIF4E expression is reduced by 4EASO treatment in mesothelioma. Cultured normal mesothelial and mesothelioma cells were transfected with mmASO or 4EASO and eIF4E and β-actin protein expression was evaluated by immunoblot analysis from lysates harvested after 72 hours. The mmASO does not alter eIF4E levels in mesothelial cells or mesothelioma cells. The band intensity levels for eIF4E following 4EASO treatment was normalized to untreated cells for each cell line and was determined using ImageJ. β-actin level does not change upon treatment and serves as a loading control.

To verify that the direct targeting of eIF4E expression with 4EASO that led to growth inhibition in mesothelioma, also led to a decrease in eIF4E protein levels, immunoblot analysis was employed. Mesothelial and mesothelioma cells were transfected with increasing doses of 4EASO or mmASO for 72 hours and lysates were prepared. The three mesothelioma cell lines exhibited extensive decreases in eIF4E protein levels that appeared to be dose dependent (Figure 2). Treatment of LP9 cells also resulted in decreased eIF4E levels quantitatively, however at these concentrations, there was very little cell death. Treatment with mmASO did not alter the eIF4E level at any of the concentrations employed. The level of β-actin was not changed during treatment with 4EASO or mmASO at all concentrations. This result is consistent with the notion that reducing eIF4E levels does not impact expression of “strong” mRNAs like that of β-actin [34]. 

### 4EASO suppression of eIF4E levels reduces assembly of cap-dependent initiation complex

**Figure 3:**
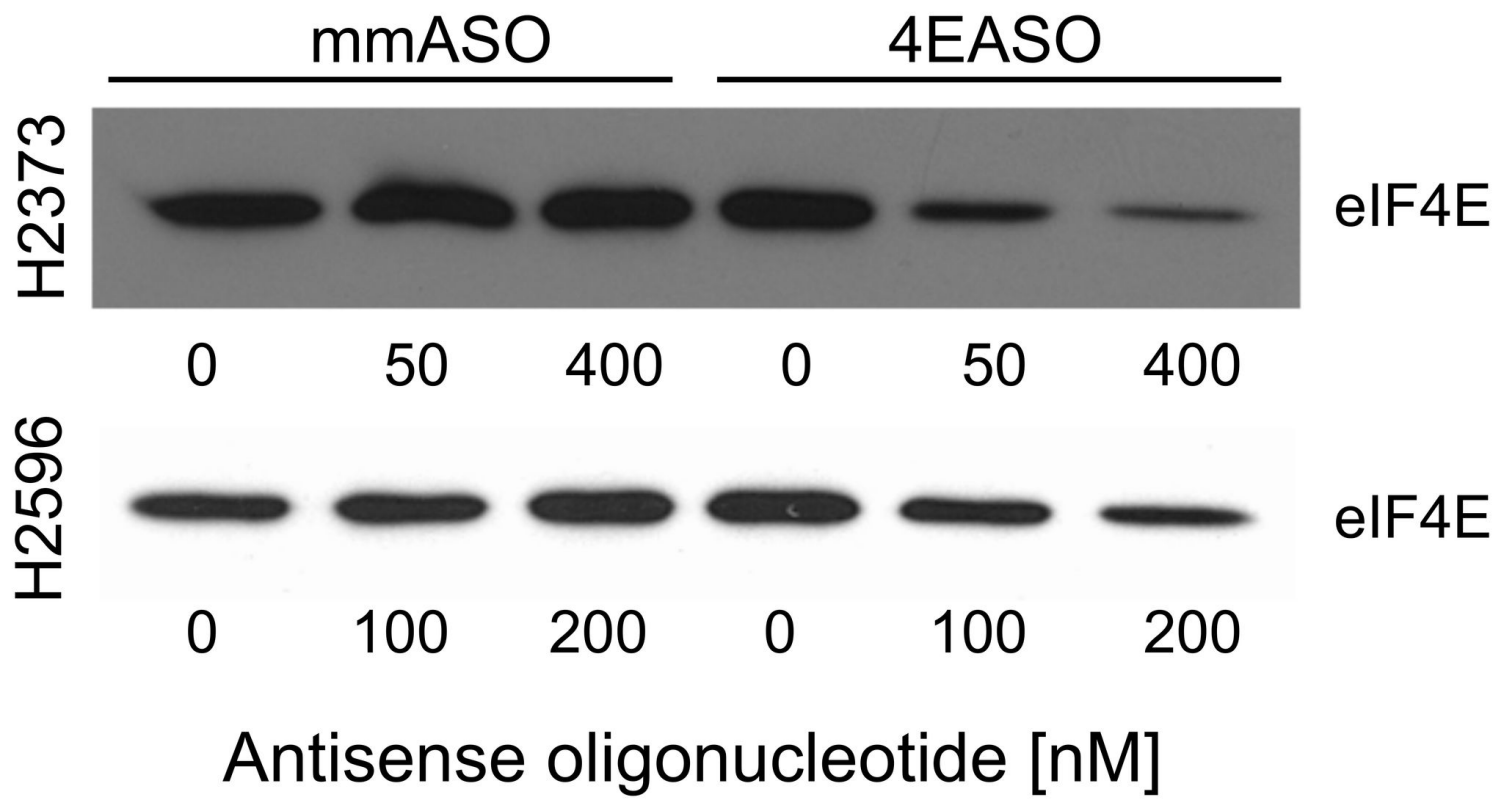
Reduced expression of eIF4E by 4EASO suppresses assembly of cap-dependent initiation complex. Mesothelioma cells were treated with 4EASO or mmASO and lysate samples were subjected to cap-analogue capture using 7^m^-GTP-sepharose before immunoblot analysis. For both mesothelioma cell lines the intensity of eIF4E to the cap-analogue was decreased in cells treated with 4EASO compared to mmASO. Transfection with mismatch control did not alter eIF4E levels bound to the cap-analogue.

To assess the impact of 4EASO mediated decrease in eIF4E levels to interfere with the assembly of the eIF4F initiation complex a cap-analogue capture of eIF4E was employed, followed by immunoblot analysis. H2373 and H2596 cells were transfected with increasing doses of 4EASO and mmASO with lysates prepared 72 hours later. These lysates were next incubated while mixing with 7-methyl-GTP-sepharose to capture eIF4E. The bound eIF4E was next eluted from the cap-analogue and immunoblot analysis used to compare eIF4E levels following treatment (Figure 3). For each cell line not transfected or transfected with mmASO eIF4E fervently bound to the cap-analogue consistent with the translationally active state. Upon treatment of each cell line with 4EASO the association of eIF4E was repressed in a dose dependent manner compared with cells not treated or treated with mmASO. The decrease in the level of eIF4E bound to the cap-analogue indicates that 4EASO strongly diminishes the levels of the eIF4F translation initiation complex.

### Apoptosis is induced in mesothelioma by 4EASO transfection

**Figure 4:**
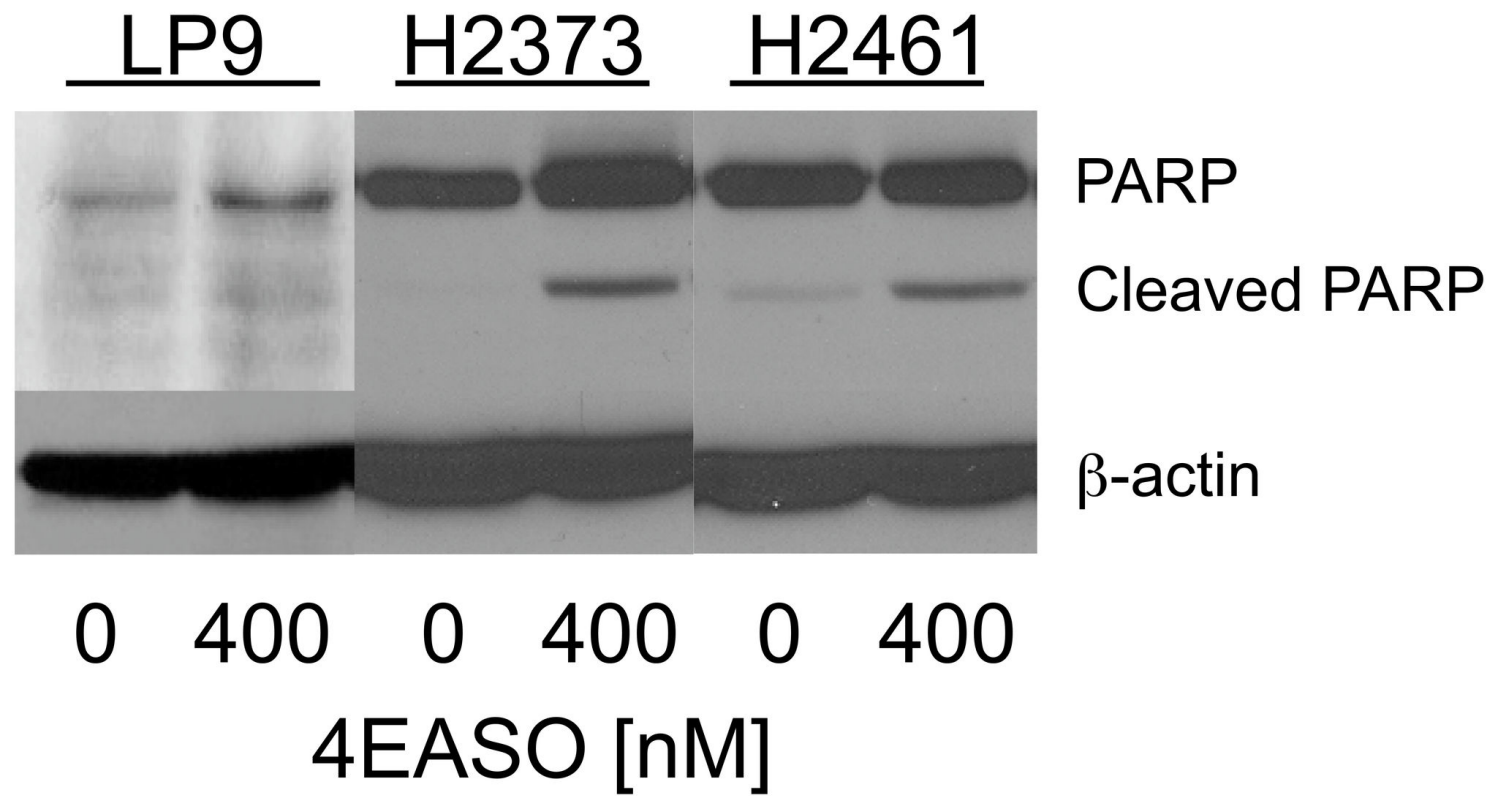
4EASO transfection induces apoptosis in mesothelioma. Mesothelioma cell lines H2373 and H2461 along with human mesothelial cells (LP9) were treated with the indicated concentration of 4EASO for 48 hours. Lysates were immunoblotted with anti-PARP antibody. In mesothelioma cell lines PARP cleavage was substantially increased in 4EASO treated compared to untreated cells.

Enforced overexpression of eIF4E can confer resistance to apoptosis [35–38]. In order to explore the possibility that suppression of eIF4E levels by 4EASO treatment would lead to apoptotic cell death in mesothelioma, poly (ADP-ribose) polymerase cleavage was investigated following 4EASO treatment. H2373, H2461 and LP9, human mesothelial cells, were not treated or treated with 4EASO and lysates were prepared 48 hours later. Immunoblot analysis revealed that 4EASO treatments increased PARP cleavage in mesothelioma cells indicating increased apoptosis compared to untreated cells. PARP cleavage appeared to be less in LP9 cells (Figure 4). 

### Treatment with 4EASO enhances susceptibility of mesothelioma cells to cytotoxic drugs

**Figure 5:**
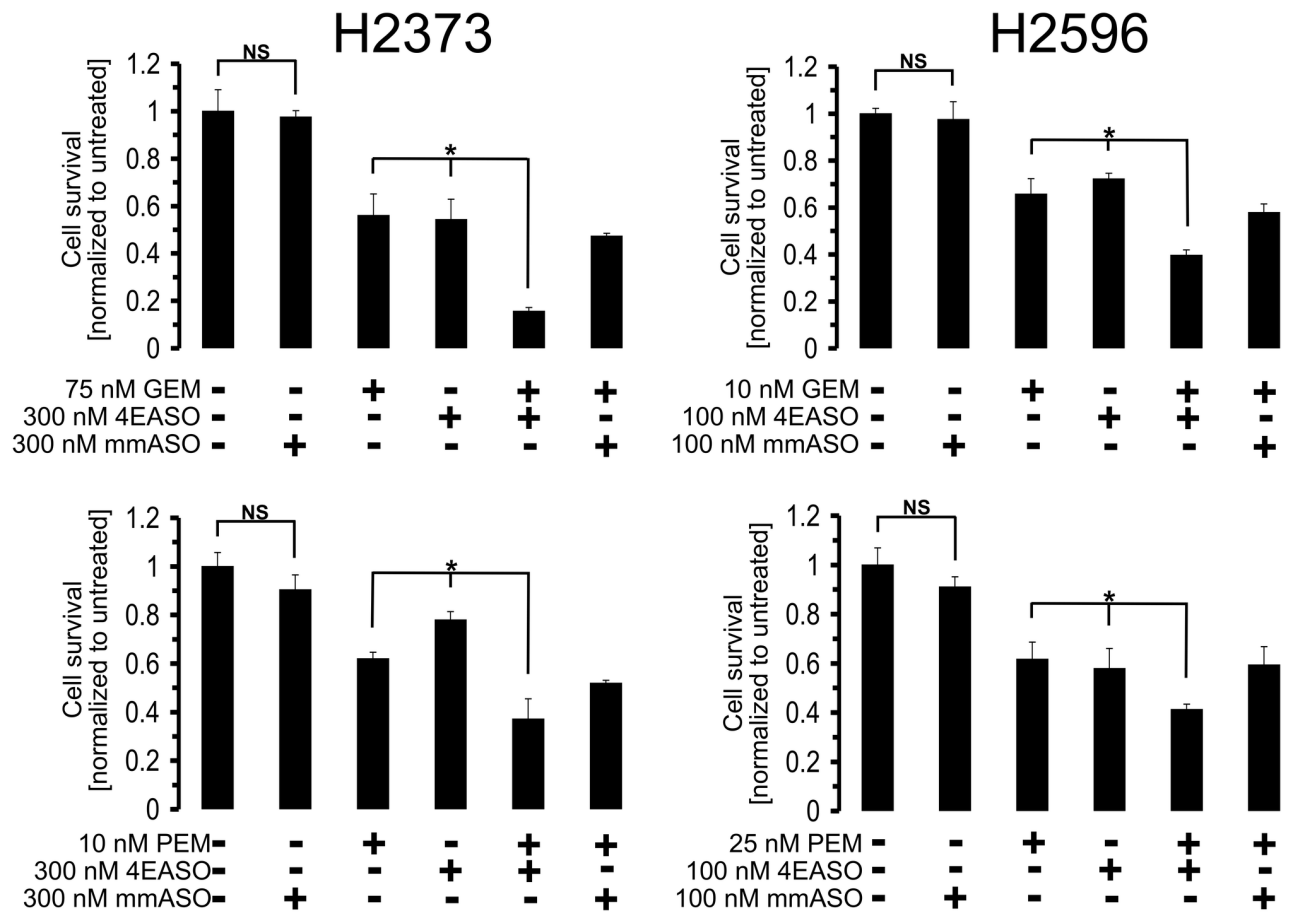
Enhanced susceptibility of mesothelioma cells treated with 4EASO to cytotoxic drugs. Mesothelioma cell lines transfected with mmASO or 4EASO were treated with the indicated concentration of gemcitabine (GEM) or pemetrexed (PEM) and viable cells were counted. Elevated cell death was found for cells treated in combination with 4EASO and pemetrexed or gemcitabine compared to each treatment alone. Concurrent treatment of mesothelioma cells with 4EASO combined with pemetrexed or gemcitabine suppresses proliferation compared to treatment with mmASO combined with pemetrexed or gemcitabine. *Columns*, the mean of three independent determinations of cell number normalized to untreated cells, *bars*, s.d. Averages of combination treatment was compared to either agent alone by Student’s *t*-test. * denotes a p value <0.05. NS = not statistically significant.

It has been reported that repression of translation initiation in cell lines of acute myelogenous leukemia [39], lung [13] and breast [40] cancers were sensitized to cytotoxic agents. To explore the possibility that 4EASO mediated suppression of translation initiation would also enhance cytotoxic induced cell killing in mesothelioma, mesothelioma cells were treated with gemcitabine and pemetrexed alone and in combination with 4EASO. Both H2373 and H2596 cell lines were treated with the indicated concentrations of gemcitabine, pemetrexed, 4EASO and mmASO (Figure 5). In both cell lines gemcitabine-induced cell death was increased when combined with 4EASO. In response to combined treatment of 4EASO plus pemetrexed treatment the cell viability decreased when compared to 4EASO treatment alone (Figure 5). There was a slight difference noted in the survival of mmASO treated cells compared to untreated cells, however, this difference was not statistically significant. Thus, the added efficacy of 4EASO to chemotherapy was more likely due to eIF4E knockdown rather than off target effects of ASO (Figure 5). Based on the results in Figure 5 we next examined the combination effects of 4EASO combined with pemetrexed or gemcitabine quantitatively using the Chou-Talalay methodology [30]. Growth inhibitory effects of 4EASO, gemcitabine, pemetrexed, alone and the combination of 4EASO and gemcitabine and 4EASO and pemetrexed were determined in cell lines H2373 and H2596 in a 96 well plate format using Cell Counting Kit-8 (Dojindo Molecular Technologies). The combination index analysis was performed using CompuSyn software and the combination indices determined (Table 1). The combination effects of 4EASO and chemotherapeutic agents were similar in that at low doses of each drug in combination yielded CI values demonstrating moderate antagonism to strong antagonism (CI>1). At higher concentrations of the two drugs the CI values obtained indicate slight to moderate synergism (CI<1) (Table 1). These results provide evidence for enhanced cell killing when 4EASO is combined with cytotoxic compounds at the most advantageous range of drug concentrations.

**Table 1:** Combination indices (CI) for the effect of 4EASO and gemcitabine or 4EASO and pemetrexed in combination treatment of H2373 and H2596 cells.

H2373			
4EASO (nM)	pemetrexed (nM)	Effect	CI
50	5	0.8307	1.1594
100	10	0.6756	0.7662
400	75	0.2977	0.9009
4EASO (nM)	gemcitabine (nM)	Effect	CI
100	10	0.8613	3.342
200	50	0.4419	0.6113
400	75	0.4014	0.8572
H2596			
4EASO (nM)	pemetrexed (nM)	Effect	CI
50	5	0.9068	1.1594
200	50	0.8121	1.1906
400	75	0.6015	1.0264
4EASO (nM)	gemcitabine (nM)	Effect	CI
50	5	0.9433	3.5975
100	10	0.9252	3.6011
200	50	0.3675	0.1299

### 4EASO suppression of eIF4E protein levels in mesothelioma cells reduces expression of malignancy-related proteins

**Figure 6:**
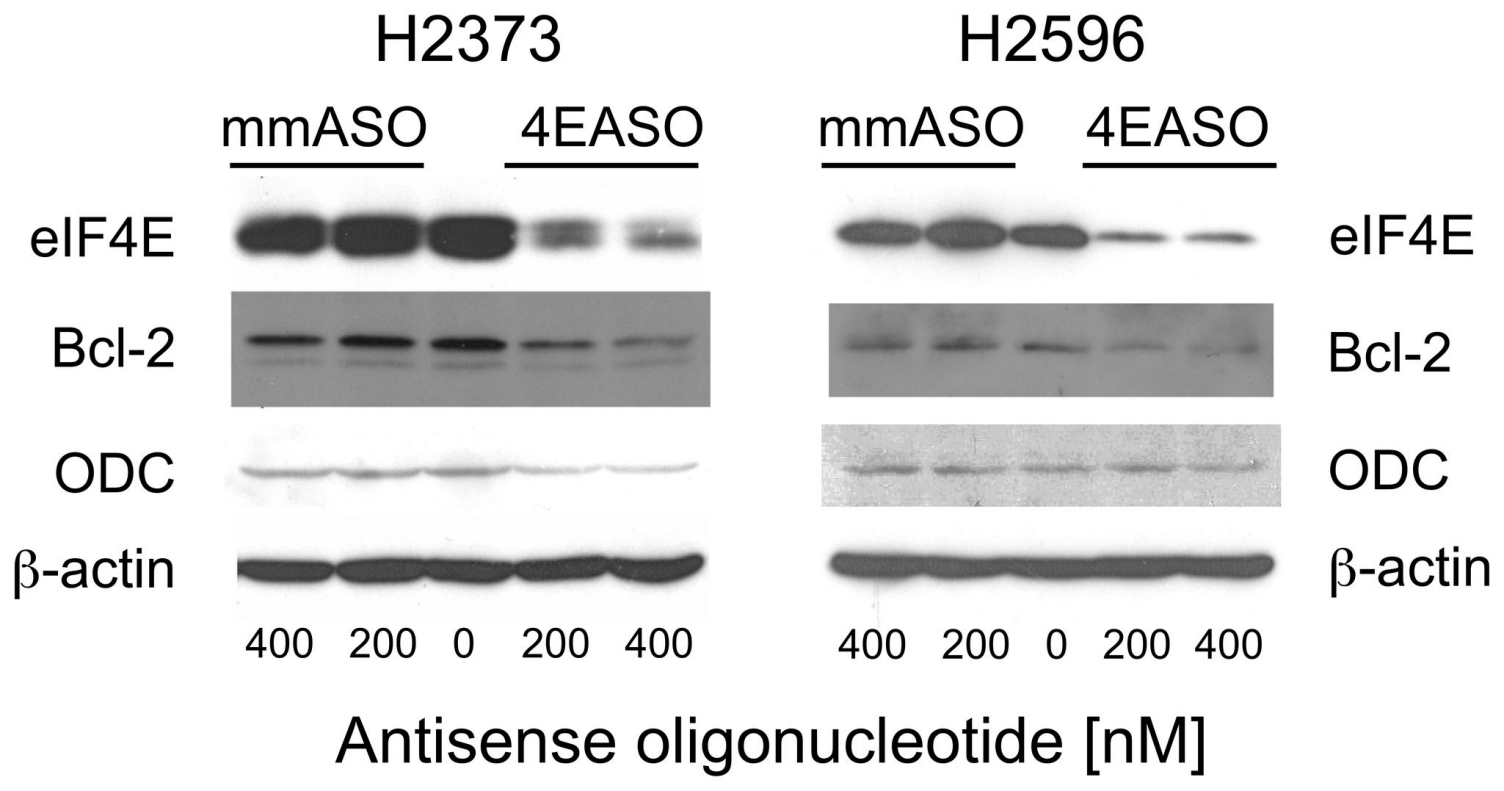
Reducing eIF4E expression in mesothelioma cells suppresses expression of malignancy related proteins. 4EASO transfection of H2373 cells resulted in reduced expression of eIF4E and eIF4E-regulated proteins Bcl-2 and ODC as demonstrated by Western analysis. eIF4E and Bcl-2 levels were diminished in H2596 cells by 4EASO treatment. Lysates were prepared 72 hours following treatment with mmASO and 4EASO. β-actin serves as a loading control.

Hyperactivation of eIF4E increases the activity of the eIF4F translation initiation complex that is believed to result in the increase in translation of a limited pool of oncogenic mRNA transcripts that drives the malignant phenotype [41]. To assess whether 4EASO mediated knockdown of eIF4E would affect the expression of malignancy-related proteins H2373 and H2596 cell lines were treated with 4EASO for 72 hours, lysates prepared and immunoblot analysis performed. Upon 4EASO treatment of both cell lines eIF4E levels were extensively diminished (Figure 6). By contrast untreated and mmASO treated cells did not result in a reduction of eIF4E protein. The anti-apoptotic protein Bcl-2 and growth regulatory protein ornithine decarboxylase (ODC) have been both shown previously to be regulated at the level of cap-dependent translation due to the extensive secondary structure of the mRNA thus requiring the eIF4F complex for efficient translation [35,42,43]. Expression of these proteins was evaluated following treatment with 4EASO and mmASO for both mesothelioma cell lines. Substantial suppression of Bcl-2 and ODC was seen in H2373 while Bcl-2 was diminished in H2596 following 4EASO treatment (Figure 6). In H2596 cells, ODC was not affected by 4EASO treatment for unclear reasons. As protein levels are dependent upon the degradation rate and mRNA expression, these factors might account for the differences seen, however, this is purely speculative. Again, β-actin expression was largely unaffected by diminished eIF4E levels, consistent with the concept that proteins with “strong” mRNAs are not selectively influenced by eIF4F activity. 

## Discussion

The view that deranged activation of cap-dependent translation is oncogenic is strongly recognized [11,44,45]. The oncogenic potential of eIF4F hyperactivity is in most instances related to the activity of eIF4E, which is the rate-limiting component of the translation initiation complex [3,4]. eIF4E overexpression is common in multiple cancer types [12–18] and clearly linked with poor clinical outcome and decreased patient survival [5]. Considerable evidence exists for therapeutic strategies that target eIF4E hyperactivation in preclinical models that result in inhibited tumor cell growth [11,13,20–27]. These studies also indicate that suppression of eIF4E leads to enhanced chemosensitivity and, in a recent investigation, to radiosensitivity [46]. 

The strategy selected here for suppressing eIF4E hyperactivity was by direct targeting of *eIF4E* mRNA by specific antisense oligonucleotide (4EASO). In previous work, the 4EASO was shown to diminish eIF4E levels and induce apoptosis in a panel of human cancer cell lines at nanomolar concentrations [12,28]. The systemic delivery of 4EASO (LY2275796) produced a knockdown in eIF4E, decreased malignancy-related proteins in a dose-dependent manner and prevented tumor growth in breast and prostate xenografts. 4EASO also targets murine *eIF4E* mRNA. While eIF4E expression was reduced in normal mouse tissues, there was no appreciable change in liver, spleen or body weight while the animals did not elicit signs of illness or distress.

These promising results led to a phase 1 single agent clinical trial of 4EASO in patients with advanced cancer. A dose escalation design was employed that determined the maximum tolerated dose (MTD) and biological effective dose (BED) of 1000 mg 4EASO. In the majority of patients a decrease in eIF4E expression was observed when comparing pre- and post treatment tumor biopsies. 4EASO is now being examined for the treatment of patients with castrate-resistance prostate cancer and for the treatment of patients with stage IV non-small cell lung cancer in combination with chemotherapy in 2 phase I/II trials [29].. While the current data show an antiproliferative effect *in vitro*, the data also support the concept of enhanced chemosensitivity with 4EASO in mesothelioma.

This current study examined 4EASO as a therapy for mesothelioma. Evidence is presented establishing that suppression of eIF4E levels induced by 4EASO treatment is correlated with reduced cell viability, induced apoptosis, disrupted eIF4F complex formation, reduced expression of malignancy related proteins and enhanced chemosensitization of mesothelioma cells. These results are expected based upon the dependence of mesothelioma cells with elevated cap-mediated translation induced by low-level expression of 4E-BP1 repressor coupled with activation of eIF4E [19]. By recapitulation of nearly normal eIF4F activity by 4EASO treatment of cancer cells, the translation of select mRNAs with long, complex 5’UTRs (i.e. those involved in malignancy) results in diminished translation to levels of normal cellular homeostasis. Further, suppression of eIF4E levels reduces translation of proteins that perform at the junction of cellular pathways that control essential functions for cancer genesis, progression and metastasis [8]. As activation of translation initiation is critical to the malignant phenotype, the therapeutic enforcement of normal translation is crucial to restore natural, benign cellular existence. In light of the findings that selectively reducing eIF4E levels substantially enhances mesothelioma cell killing by either pemetrexed or gemcitabine support the clinical evaluation of 4EASO combined with chemotherapy. 
